# Whole genome sequencing and *in vitro* activity data of *Escherichia* phage NTEC3 against multidrug-resistant Uropathogenic and extensively drug-resistant Uropathogenic *E. coli* isolates

**DOI:** 10.1016/j.dib.2022.108479

**Published:** 2022-07-18

**Authors:** Naveen Chaudhary, Dharminder Singh, Ravi Kumar Maurya, Balvinder Mohan, Ravimohan S. Mavuduru, Neelam Taneja

**Affiliations:** a Department of Medical Microbiology, Postgraduate Institute of Medical Education and Research, Chandigarh, India; b Urology, Postgraduate Institute of Medical Education and Research, Chandigarh, India

**Keywords:** Phage, Multidrug-resistant, Uropathogenic *Escherichia coli*, Urinary tract infections, Sequencing, Biofilms

## Abstract

This data article describes the whole-genome sequencing and *in vitro* activity data of *Escherichia* phage NTEC3 isolated from a community sewage sample in Chandigarh, India. The phage NTEC3 was active against multi-drug-resistant (MDR) and extensively drug-resistant (XDR) biofilm-forming Uropathogenic *Escherichia coli* (UPEC) strains. The genome of this phage was linear, double-stranded, and 44.2 kb long in size. A total of 72 ORFs (open reading frames) were predicted and 30 ORFs were encoded for functional proteins. The phage belonged to the *Kagunavirus* genus of the *Siphoviridae* family. Phylogenetic analysis using DNA polymerase was performed to understand the phage evolutionary relationships. Genes encoding for lysogeny, virulence, toxins, antibiotic resistance, and the CRISPR/CRISPR-like system were not found during screening. The annotated genome was deposited in Genbank under the accession number OK539620.

**Specifications Table**

**Table:** 

Subject	Microbiology
Specific subject area	Medical microbiology
Type of data	WGS and *in vitro* testing data are provided in tables and figures.
How the data were acquired	WGS data were generated using Illumina Novaseq 6000 platform
Data format	Analyzed
Description of data collection	The phage NTEC3 was isolated from a community sewage water in Chandigarh, India. DNA was extracted and WGS has performed on Illumina Novaseq 6000 sequencer with a paired-end library of a read length of 2 × 150 bp [1,2]. The genome was assembled using the strategic k-mer extension for scrupulous assemblies (SKESA v2.4.0) assembler with default k-mer sizes. The ORFs were predicted using a gene locator and interpolated markov modeler (GLIMMER v3.02) and GeneMarkS v4.28 and subsequently annotated with PHASTER and RAST servers.
Data source location	Institution: Postgraduate Institute of Medical Education and Research City/Town/Region: Chandigarh Country: India Latitude and longitude for collected samples/data: 30.7650° N, 76.7750° E
Data accessibility	Repository name: GenBank, Sequence Reads Archive (SRA) Data identification numbers: OK539620, SRP369723 The direct URL to the data is as follows: https://www.ncbi.nlm.nih.gov/nuccore/OK539620 https://trace.ncbi.nlm.nih.gov/Traces/index.html?view=study&acc=SRP369723

**Value of the Data**

• Data provides genomic information on a lytic phage to researchers for sequence comparison and evolutionary relationship studies.
• Genome sequencing and *in vitro* activity data of the phage can be used by the scientific community for screening and identification of novel phage-based antimicrobial strategies.
• Data provides information about a potentially safe product for therapeutics against drug-resistant UPEC as the phage lacked genes encoding for lysogeny, virulence, toxins, and antibiotic resistance.
• Data provides information about a lytic enzyme endolysin whose sequence could be used to design recombinant endolysins to treat biofilm-associated infections.

## 1 Data Description

UPEC strains have a variety of virulence-associated factors (VFs) like adhesins, toxins, siderophores, chaperone-usher (CU) fibers, invasins, and serum resistance-associated proteins that help to invade and injure the host [3].

Many UTIs causing UPEC strains carry different antibiotic resistance genes like Extended-Spectrum-ß-Lactamases (ESBLs) and Metallo-ß-Lactamases (MBLs) on their chromosomes. UTIs caused by MDR and XDR UPECs account for one of the major therapeutic challenges in the health sector [4]. The widespread decline in antibiotic effectiveness has sparked renewed interest in alternative therapeutics like phage therapy. Phage therapy is primarily based on the use of obligately lytic phages to eliminate their bacterial hosts while leaving human cells unharmed.

**Fig. 1:**
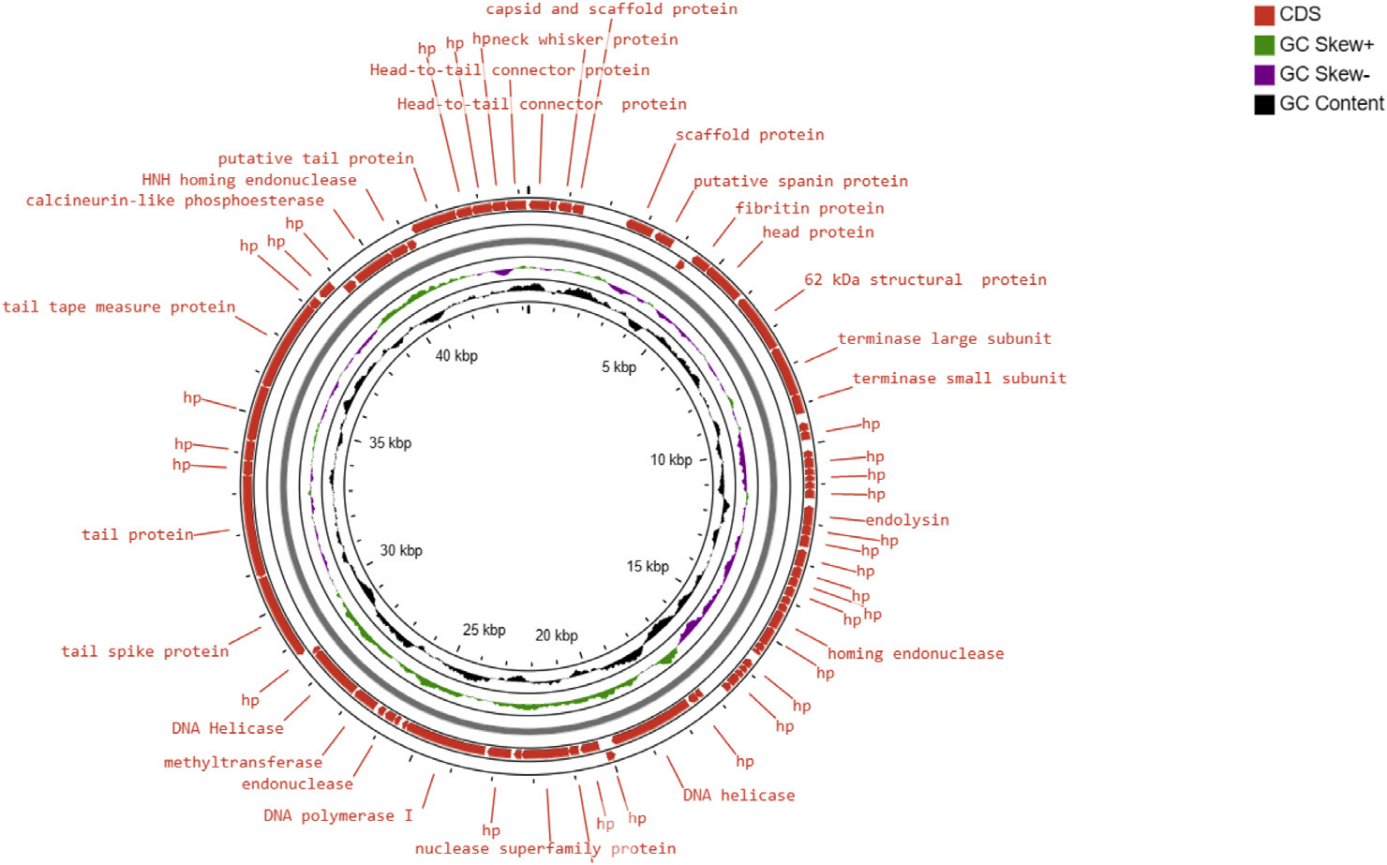
Circular genome view of phage NTEC3 constructed using CGView.

**Fig. 2:**
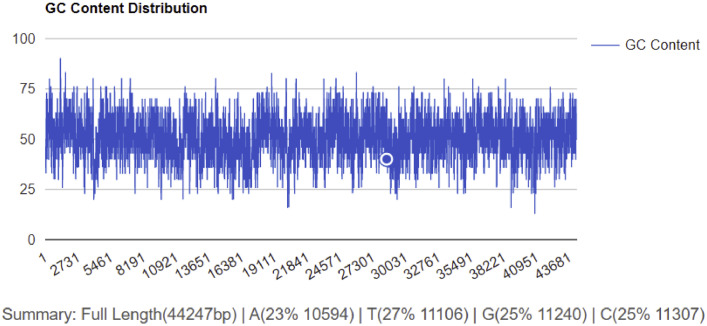
GC content distribution pattern of phage NTEC3.

**Fig. 3:**
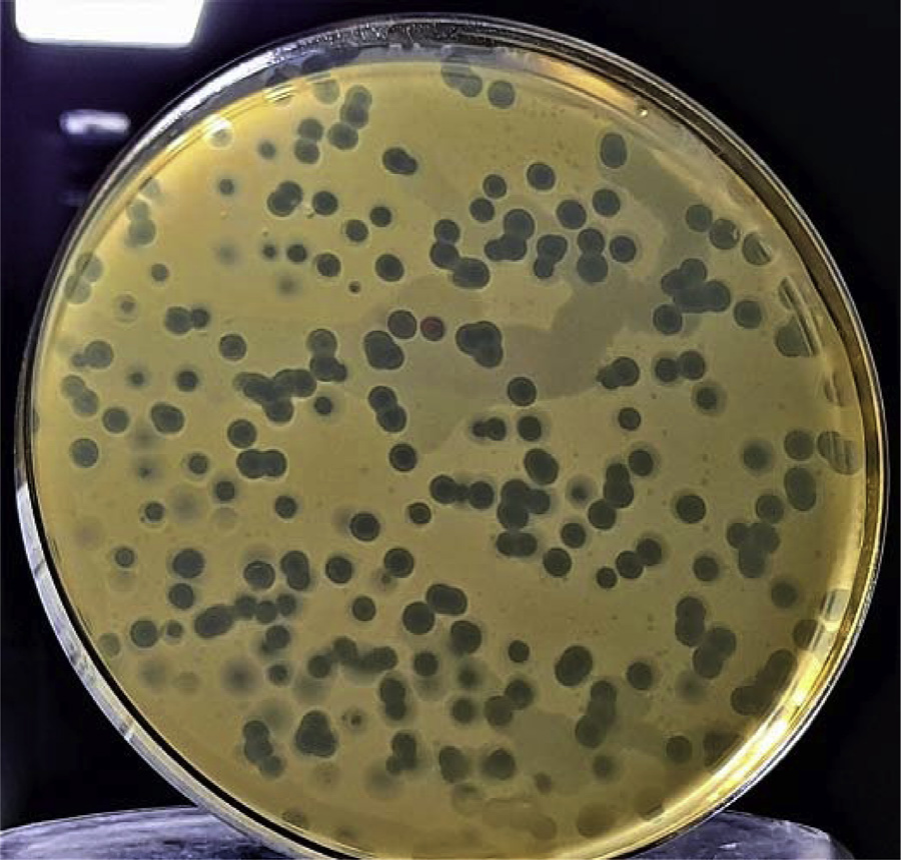
Plaque morphology of phage NTEC3.

**Fig. 4:**
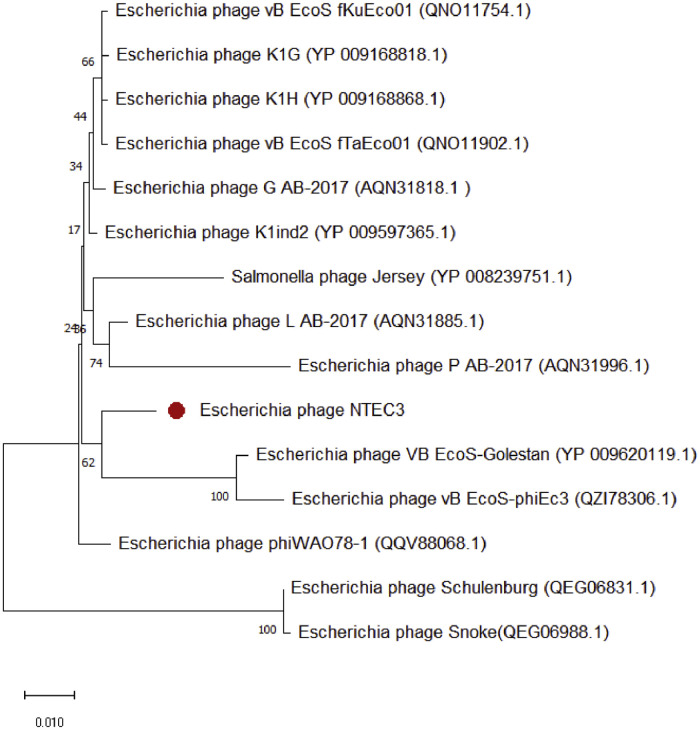
The neighbor-joining tree constructed based on the amino acid sequence of DNA polymerase protein using MEGA-X. Bootstraps values in the percentage of 1000 replicates are depicted next to the branches.

Phage NTEC3 was isolated from community sewage water in Chandigarh, India using a clinical strain UPEC 590B as a host bacterium. The NEBNext Ultra kit was used to prepare the sequencing library, and sequencing was performed on Novaseq 6000 sequencer, yielding 6974384 paired-end (150-bp-long) raw reads (Table 1). The complete genome of the phage was 44.2 kb in length, with a gene density of 1.60/kbp, and a GC content of 51% (Table 1, Fig. 1, Fig. 2). Out of the 72 predicted ORFs, 21 (27.3%) were found to be present in the direct strand, and the rest were found in the complementary strand (Fig. 1). Forty-four ORFs (61.6%) were predicted to encode for hypothetical proteins whereas 30 ORFs (41 %) were predicted to encode for functional proteins. The functional proteins were categorized into the following groups (i) DNA replication/metabolism-related proteins (ii) Host lysis and adhesion-related proteins (iii) DNA packaging proteins (iv) Structural proteins (Table S1). The genome of this phage lacked genes encoded for temperate phage markers, toxins, virulence factors, antibiotic resistance, or the CRISPR/CRISPR-like system. The ORF 22 of phage NTEC3 encoded for an endolysin that has an intrinsic feature to disrupt biofilm mass and bacterial cell lysis by breaking the peptidoglycan layer [5]. Phage NTEC3 formed plaques of 4-5 mm in diameter (Fig. 3). The phage was active against 24.4% of 45 MDR and XDR UPEC strains resistant to third-generation cephalosporins, fluoroquinolones, aminoglycosides, beta-lactamase inhibitor combinations, cotrimoxazole, nitrofurantoin and imipenem (Table S2). In the phylogenetic tree, phage NTEC3 was placed in an outgroup of two *Siphoviridae* family phages *Escherichia* phage VB_EcoS-Golestan (BLASTP identity >93.18%) and *Escherichia* phage vB_EcoS-phiEc3 (BLASTP identity >92.78%) isolated from wastewater samples in USA and Spain, respectively (Fig. 4).

**Table 1:** Genome sequence characteristic of phage NTEC3.

Index	Value
Raw reads generated	6974384
Read length	150 bp
Library type	Paired-end
Genome size	44240
GC content	51%
Number of predicted genes with significant BLASTX match (E-value <=1e-3 and Similarity score >=40%) with uniprot	72
Accession no.	OK539620
No. of Lysis /adhesion-related proteins	2
Number of tRNAs	0
CRISPR-cas sequence	0

## 2 Experimental Design, Materials and Methods

**Fig. 5:**
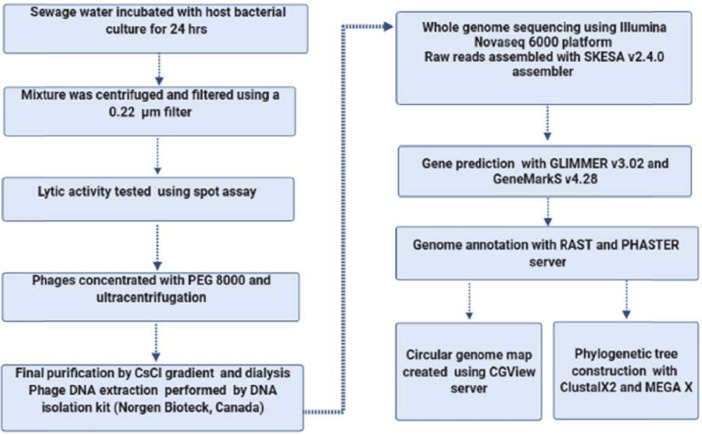
Flow chart showing the experimental design protocol used for phage isolation and sequencing.

A flow chart was designed using Cmap server to demonstrate the experimental design and methods (Fig. 5) [6,7].

### 2.1 Phage Isolation

The *Escherichia* phage NTEC3 was isolated from the community sewage treatment plant in Chandigarh. MDR UPEC 590 strain was used as host bacterium to amplify and propagate phage NTEC3. The raw sewage water samples were centrifuged at 1500 RPM and the supernatant was filtered through a 0.45 µm membrane filter. The filtrate was incubated at 37°C with different bacterial cultures for 18 h. After incubation, the mixture was centrifuged at 4000 RPM and filtered using a 0.22 µm syringe filter. The lytic activity of the filtrate was evaluated using a spot assay against the respective bacterial strains.

### 2.2 Phage Purification

A clear spot on agar was scrapped and incubated with the host bacterial culture for overnight incubation at 37°C. A single isolated plaque was picked after repeating the plaque assay experiment thrice. Phage plaque size was measured and expressed in millimeters. Phages were concentrated using the overnight polyethylene glycol (PEG 8000) precipitation method followed by ultracentrifugation (50,000 RPM) and the ultrafiltration method [8]. Final purification was performed with CsCl gradient and dialyzed against SM buffer (pH 7.5) using tubing membrane (MWCO 12,000). Host range activity of phage NTEC3 was tested against 45 MDR and XDR UPEC strains using the standardized spot assay method.

### 2.3 Genome Sequencing and Analysis

The DNA extraction was performed with a phage DNA isolation kit (Norgen Bioteck, Canada). The whole genome of the phage was sequenced using the Illumina Novaseq 6000 platform with a paired-end library of a read length of 2 × 150 bp [9].

SKESA v2.4.0 assembler was used to execute de novo assembly with default k-mer sizes [10]. GLIMMER v3.02 and GeneMarkS v4.28 were used to predict genes from the assembled sequence and annotated with PHASTER and RAST server and also searched against BLASTP in the UniProt database [11], [12], [13]. The CGView server was used to make a circular genomic map of the phage genome [14]. The amino acid sequences of the DNA polymerase gene of similar phages with BLASTp identity of >90% were used for constructing the phylogenetic tree.

## Ethics Statements

This study was approved by the Institute Ethical Clearance Committee of the postgraduate institute of Medical Education and Research (Chandigarh, India).

## CRediT authorship contribution statement

**Naveen Chaudhary:** Methodology, Software, Data curation, Writing – original draft. **Dharminder Singh:** Methodology, Data curation. **Ravi Kumar Maurya:** Methodology, Data curation. **Balvinder Mohan:** Supervision, Validation, Data curation. **Ravimohan S. Mavuduru:** Supervision, Validation, Data curation. **Neelam Taneja:** Supervision, Resources, Conceptualization, Validation, Writing – review & editing.

## Declaration of Competing Interest

The authors declare that they have no known competing financial interests or personal relationships that could have appeared to influence the work reported in this paper.

## Data Availability

SRA accession number (Original data) (SRA). GenBank accession number (Original data) (GenBank).

## Appendix Supplementary materials

Image, application 1

## References

[1] Chaudhary N., Singh D., Narayan C., Samui B., Mohan B., Mavuduru R.S., Taneja N. (2021). Complete genome sequence of escherichia phage 590B, active against an extensively drug-resistant Uropathogenic Escherichia coli isolate. Microbiol. Resour. Announc..

[2] Chaudhary N., Mohan B., Taneja N. (2018). Draft genome sequence of Escherichia phage PGN829.1, active against highly drug-resistant Uropathogenic Escherichia coli. Microbiol. Resour. Announc..

[3] Behzadi P. (2020). Classical chaperone-usher (CU) adhesive fimbriome: Uropathogenic Escherichia coli (UPEC) and urinary tract infections (UTIs). Folia Microbiol..

[4] Behzadi P., García-Perdomo H.A., Karpiński T.M. (2020). Metallo-ß-lactamases: a review. Mol. Biol. Rep..

[5] Schmelcher M., Donovan D.M., Loessner M.J. (2012). Bacteriophage endolysins as novel antimicrobials. Future Microbiol..

[6] Behzadi P., Gajdács M. (2021). Writing a strong scientific paper in medicine and the biomedical sciences: a checklist and recommendations for early career researchers. Biol. Future.

[7] Ranjbar R., Behzadi P., Najafi A., Roudi R. (2017). DNA microarray for rapid detection and identification of food and water borne bacteria: from dry to wet lab. Open Microbiol. J..

[8] Hietala V., Horsma-Heikkinen J., Carron A., Skurnik M., Kiljunen S. (2019). The removal of endo- and enterotoxins from bacteriophage preparations. Front. Microbiol..

[9] Senabouth A., Andersen S., Shi Q., Shi L., Jiang F., Zhang W., Wing K., Daniszewski M., Lukowski S.W., Hung S.S.C., Nguyen Q., Fink L., Beckhouse A., Pébay A., Hewitt A.W., Powell J.E. (2020). Comparative performance of the BGI and Illumina sequencing technology for single-cell RNA-sequencing. NAR Genom. Bioinf..

[10] Souvorov A., Agarwala R., Lipman D.J. (2018). SKESA: strategic k-mer extension for scrupulous assemblies. Genom. Biol..

[11] Arndt D., Grant J.R., Marcu A., Sajed T., Pon A., Liang Y., Wishart D.S. (2016). PHASTER: a better, faster version of the PHAST phage search tool. Nucleic Acids Res..

[12] Aziz R.K., Bartels D., Best A., DeJongh M., Disz T., Edwards R.A., Formsma K., Gerdes S., Glass E.M., Kubal M., Meyer F., Olsen G.J., Olson R., Osterman A.L., Overbeek R.A., McNeil L.K., Paarmann D., Paczian T., Parrello B., Pusch G.D., Reich C., Stevens R., Vassieva O., Vonstein V., Wilke A., Zagnitko O. (2008). The RAST server: rapid annotations using subsystems technology. BMC Genom..

[13] Delcher A.L., Bratke K.A., Powers E.C., Salzberg S.L. (2007). Identifying bacterial genes and endosymbiont DNA with glimmer. Bioinformatics.

[14] Grant J.R., Stothard P. (2008). The CGView server: a comparative genomics tool for circular genomes. Nucleic Acids Res..

