# Supplementary material for: Whole genome sequencing and in vitro activity data of Escherichia phage NTEC3 against multidrug-resistant Uropathogenic and extensively drug-resistant Uropathogenic E. coli isolates
Source: Data Brief. 2022 Jul 18;43:108479. doi: 10.1016/j.dib.2022.108479 (PMC9340528; doi:10.1016/j.dib.2022.108479)
Supplement: Supplementary file 1 [file mmc1.doc]

**Supplementary Tables**

**Table S1: Functional annotation of phage NTEC3**

| ORF | Start | Stop | Directions | No. of residues | Predicted function | Identity (%) | | Color code |
| --- | --- | --- | --- | --- | --- | --- | --- | --- |
| 1 | 513 | 1 | - | 170 | Head-to-tail connector complex protein | 98.2 (Escherichia phage VB_EcoS-Golestan) | |  |
| 2 | 696 | 517 | - | 59 | Hypothetical protein | 96.6 (*Escherichia* phage vB_EcoS_XY1) | |  |
| 3 | 1074 | 733 | - | 113 | Phage neck whisker | 93.3 (*Escherichia* phage vB_EcoS_XY2) | |  |
| 4 | 1386 | 1087 | - | 128 | Capsid and scaffold protein | 99.0 (*Escherichia* phage vB_EcoS_XY2) | |  |
| 5 | 3199 | 2498 | - | 233 | Scaffold protein | 100 (*Escherichia* phage vB_EcoS_XY1) | |  |
| 6 | 3777 | 3391 | - | 147 | Putative spanin protein | 95.3 (Escherichia phage vB_EcoS-Ro145clw) | |  |
| 7 | 4153 | 4356 | + | 67 | Hypothetical protein | 43.9 (Salmonella phage Shemara) | |  |
| 8 | 4847 | 4836 | - | 183 | Fibritin protein | 98 (Escherichia phage ZCEC5) | |  |
| 9 | 5890 | 4847 | - | 347 | Head protein | 98.3 (Escherichia phage vB_EcoS_XY2) | |  |
| 10 | 7441 | 5960 | - | 520 | 62 kDa structural protein | 100 (Escherichia phage vB_EcoS_XY2) | |  |
| 11 | 8725 | 7454 | - | 423 | Terminase large subunit | 100 (Escherichia phage vB_EcoS_XY2) | |  |
| 12 | 9224 | 8715 | - | 169 | Terminase small subunit | 99.4 ([*Escherichia* phage vB_EcoS_XY2](https://www.uniprot.org/taxonomy/2681675)) | |  |
| 13 | 9444 | 9346 | - | 79 | Hypothetical protein | 87.9 **(**[*Escherichia* phage vB_EcoS_fKuEco01](https://www.uniprot.org/taxonomy/2762428)**)** | |  |
| 14 | 9659 | 9441 | - | 98 | Hypothetical protein | 37 ([*Proteus* phage vB_PmiP_RS51pmB](https://www.uniprot.org/taxonomy/2250313)**)** | |  |
| 15 | 9892 | 9656 | - | 97 | Hypothetical protein | 100 ([Escherichia phage vB_EcoS_HSE2](https://www.uniprot.org/taxonomy/2053690)) | |  |
| 16 | 10059 | 9889 | - | 56 | Hypothetical protein | 100 ([*Escherichia* phage vB_EcoS_fKuEco01](https://www.uniprot.org/taxonomy/2762428)) | |  |
| 17 | 10329 | 10147 | - | 60 | Hypothetical protein | 98.3 (*Escherichia* phage vB_EcoS_XY2) | |  |
| 18 | 10594 | 10316 | - | 118 | Hypothetical protein | 82.8 (*Escherichia* phage vB_EcoS_XY2) | |  |
| 19 | 10772 | 10587 | - | 61 | Hypothetical protein | 100 ([Escherichia phage VB_EcoS-Golestan](https://www.uniprot.org/taxonomy/2047801)**)** | |  |
| 20 | 10926 | 10765 | - | 74 | Hypothetical protein | 98.1 (*Escherichia* phage vB_EcoS_XY2) | |  |
| 21 | 11359 | 11111 | - | 82 | Head protein | 97.6 ([*Escherichia* phage VB_EcoS-Golestan](https://www.uniprot.org/taxonomy/2047801)**)** | |  |
| 22 | 12028 | 11540 | - | 162 | Endolysin | 95.1(Escherichia phage vB_EcoS_fKuEco01) | |  |
| 23 | 12278 | 12006 | - | 116 | Hypothetical protein | 98.9(*Raoultella* phage RP180) | |  |
| 24 | 12579 | 12271 | - | 102 | Hypothetical protein | 99 ([*Escherichia* phage vB_EcoS-Ro145clw](https://www.uniprot.org/taxonomy/2079541)) | |  |
| 25 | 13076 | 12639 | - | 145 | Hypothetical protein | 89 (*Raoultella* phage RP180) | |  |
| 26 | 13366 | 13073 | - | 97 | Hypothetical protein | 99 (*Escherichia* phage vB_EcoS_XY2) | |  |
| 27 | 13601 | 13350 | - | 83 | Hypothetical protein | 74 ([*Escherichia* phage vB_EcoS_fKuEco01](https://www.uniprot.org/taxonomy/2762428)) | |  |
| 28 | 14073 | 13870 | - | 89 | Hypothetical protein | 100 (*Escherichia* phage vB_EcoS_XY2) | |  |
| 29 | 14278 | 14075 | - | 88 | Hypothetical protein | 98.5(Escherichia phage P AB-2017) | |  |
| 30 | 14748 | 14275 | - | 197 | Homing endonuclease | 50.3 ( (*Salmonella* phage Shemara) | |  |
| 31 | 15212 | 14745 | - | 181 | Hypothetical protein | *72.5 (*Escherichia phage vB_EcoS_fKuEco01*)* | |  |
| 32 | 15397 | 15209 | - | 62 | Hypothetical protein | *90.3 (*Escherichia phage vB_EcoS_fFiEco02*)* | |  |
| 33 | 15515 | 15387 | - | 42 | Hypothetical protein | *100 (*Escherichia phage vB_EcoS_fTaEco01*)* | |  |
| 34 | 15923 | 15723 | - | 66 | Hypothetical protein | *98.5 (*Escherichia phage vB_EcoS_fKuEco01*)* | |  |
| 35 | 16071 | 15916 | - | 51 | Hypothetical protein | 100 (*Escherichia* phage vB_EcoS_fKuEco01*)* | |  |
| 36 | 16223 | 16068 | - | 51 | Hypothetical protein | *98 (*Escherichia phage G AB-2017*)* | |  |
| 37 | 16513 | 16220 | - | 97 | Hypothetical protein | 73.9 (Escherichia phage vB_EcoS_XY1) | |  |
| 38 | 16704 | 16513 | - | 63 | Hypothetical protein | 93 (*Escherichia* phage vB_EcoS_XY2) | |  |
| 39 | 16807 | 17022 | + | 71 | Hypothetical protein | 89 (*Siphoviridae* sp.) | |  |
| 40 | 17200 | 17370 | + | 56 | Hypothetical protein | 100 (*Escherichia* phage P AB-2017) | |  |
| 41 | 17367 | 17600 | + | 77 | Hypothetical protein | 100 (*Escherichia* phage ZCEC5) | |  |
| 42 | 17660 | 19900 | + | 746 | DNA helicase | 96 (Escherichia phage vB_EcoS_XY1) | |  |
| 43 | 20133 | 19918 | - | 71 | Hypothetical protein | *100 (*Escherichia phage G AB-2017*)* | |  |
| 44 | 20258 | 20758 | + | 166 | Hypothetical protein | *98.2 (*Escherichia phage VB_EcoS-Golestan*)* | |  |
| 45 | 20802 | 21074 | + | 116 | Hypothetical protein | 91.1(Raoultella phage RP180) | |  |
| 46 | 21071 | 22312 | + | 413 | Nuclease superfamily protein | 96.6 (*Escherichia* phage G AB-2017) | |  |
| 47 | 22309 | 22512 | + | 67 | Hypothetical protein | 79.1(*Escherichia* phage ZCEC5) | |  |
| 48 | 22586 | 23212 | + | 208 | Hypothetical protein | 99.5 (*Escherichia* phage K1G) | |  |
| 49 | 23272 | 25452 | + | 726 | DNA Polymerase 1 | 98.2 (*Escherichia* phage G AB-2017) | |  |
| 50 | 25442 | 25573 | + | 69 | Hypothetical protein | 100 (*Escherichia* phage vB_EcoS_XY2) | |  |
| 51 | 25656 | 25808 | + | 50 | Hypothetical protein | 100 (*Escherichia* phage vB_EcoS_XY2) | |  |
| 52 | 25805 | 26089 | + | 113 | Endonuclease protein | 97.9 (Escherichia phage vB_EcoS_fFiEco02) | |  |
| 53 | 26120 | 26311 | + | 63 | Hypothetical protein | 98.4(S*almonella* phage SS1) | |  |
| 54 | 26396 | 27058 | + | 220 | Methyl transferase | *82.2 (*Escherichia phage vB_EcoS_fKuEco01*)* | |  |
| 55 | 27095 | 28519 | + | 474 | DNA helicase | *98.1 (*Escherichia phage vB_EcoS_fTaEco01*)* | |  |
| 56 | 28516 | 28653 | + | 45 | Hypothetical protein | 100 (*Escherichia* phage P AB-2017) | |  |
| 57 | 30862 | 28682 | - | 726 | Spike protein | 93.7(*Escherichia* phage vB_EcoS_XY2) | |  |
| 58 | 33442 | 30875 | - | 855 | Tail protein | *98.5 (*Escherichia phage VB_EcoS-Golestan) | |  |
| 59 | 33798 | 33433 | - | 121 | Hypothetical protein | 100 (*Escherichia* phage K1G) | |  |
| 60 | 34310 | 33795 | - | 116 | Hypothetical protein | 99.4(*Escherichia* phage ZCEC5) | |  |
| 61 | 35714 | 34314 | - | 466 | Hypothetical protein | 98.1(*Escherichia* phage vB_EcoS_XY2) | |  |
| 62 | 38026 | 35714 | - | 770 | Tail tape measure protein | 99 (*Escherichia* phage vB_EcoS_XY2) | |  |
| 63 | 38291 | 38019 | - | 90 | Hypothetical protein | 98.9 (*Escherichia* phage K1H) | |  |
| 64 | 38797 | 38381 | - | 138 | Hypothetical protein | 100 (*Escherichia* phage K1H) | |  |
| 65 | 38962 | 39288 | + | 108 | Hypothetical protein | 96.3 (*Escherichia* phage vB_EcoS_XY2) | |  |
| 66 | 39354 | 40484 | + | 376 | Calcineurin-like phosphoterase superfamily domain protein | *97.9 (*Escherichia phage vB_EcoS-Ro145clw) | |  |
| 67 | 40481 | 40951 | + | 195 | HNH homing endonuclease | 98.7 *(*Escherichia phage vB_EcotS-Ro145clw) | |  |
| 68 | 40965 | 41195 | - | 76 | Hypothetical protein | 100 (*Escherichia* phage vB_EcoS_HSE2) | |  |
| 69 | 42390 | 41224 | - | 388 | Putative tail protein | 99 (*Escherichia* phage K1H) | |  |
| 70 | 42806 | 42393 | - | 137 | Tail protein | 100 (*Escherichia* phage vB_EcoS_XY2) | |  |
| 71 | 43306 | 42806 | - | 166 | Tail protein | 100 (*Escherichia* phage vB_EcoS_XY2) | |  |
| 72 | 43662 | 43303 | - | 119 | Head-to-tail connector | 95 (*Escherichia* phage vB_EcoS_XY2) | |  |
|  | | | | | |  | Adhesion and Lysis proteins | |
|  |  |  |  |  |  |  | Structural proteins | |
|  |  |  |  |  |  |  | Replication/metabolism proteins | |
|  |  |  |  |  |  |  | Hypothetical proteins | |

**Table S2: Antibiotic sensitivity profile of 45 MDR Uropathogenic *E. coli* panel strains and host range testing of phage NTEC3 using spot assay**

| **Sr .No.** | Strain name | **Cefotaxime** | **Cefoperazone** | **Gentamycin** | **Amikacin** | **Nalidixic acid** | **Norfloxacin** | **Ciprofloxacin** | **Nitrofurantoin** | **Imipenem** | **Cotrimoxazole** | **Cefoperazone+ sulbactam**  **sulbactam** | **Colistin** | **Phage NTEC3 activity** |
| --- | --- | --- | --- | --- | --- | --- | --- | --- | --- | --- | --- | --- | --- | --- |
| 1 | 30608 | R | R | R | R | R | R | R | S | I | R | R | S | - |
| 2 | 43836 | R | R | R | R | R | R | R | S | R | R | R | S | - |
| 3 | 590 | R | R | R | R | R | R | R | S | I | R | R | S | + |
| 4 | 40329 | S | R | S | R | R | S | R | S | I | R | R | S | - |
| 5 | 38036 | R | R | R | R | R | R | R | R | I | R | R | R | - |
| 6 | 15286 | R | R | R | R | R | R | R | S | R | R | R | S | - |
| 7 | 24331 | R | R | R | R | R | R | R | I | R | R | R | S | - |
| 8 | 33448 | R | R | R | R | R | R | R | R | I | R | R | R | - |
| 9 | 12407 | R | R | R | R | R | R | R | S | R | R | R | S | + |
| 10 | 43649 | S | R | S | R | R | R | R | R | S | R | R | S | - |
| 11 | 4183 | R | R | S | S | R | R | R | S | R | R | R | S | - |
| 12 | 3919 | R | R | R | R | R | R | R | R | R | R | R | S | - |
| 13 | 34449 | R | R | R | R | R | R | R | R | S | S | R | S | - |
| 14 | 38807 | R | R | R | R | R | R | R | S | S | R | R | S | - |
| 15 | 44649 | S | R | R | R | R | R | R | R | S | R | R | S | - |
| 16 | 42829 | R | R | R | R | R | R | R | R | R | R | R | R | - |
| 17 | 39854 | R | R | R | R | R | R | R | R | S | S | R | S | - |
| 18 | 42307 | R | R | R | S | R | R | R | S | S | R | R | S | - |
| 19 | 8824 | R | S | R | S | R | R | R | S | I | R | S | S | + |
| 20 | 43839 | R | R | R | R | R | R | R | R | R | S | S | S | - |
| 21 | 1195 | R | R | S | S | R | R | R | S | S | R | R | S |  |
| 22 | 22044 | R | R | R | R | R | R | R | S | R | R | R | S | - |
| 23 | 40137 | R | R | R | R | R | R | R | R | R | R | R | R | - |
| 24 | 38929 | S | R | R | R | R | S | R | S | R | R | R | S | - |
| 25 | 40354 | R | S | R | S | R | R | R | S | I | R | S | S | - |
| 26 | 39314 | R | R | R | S | R | R | R | R | S | R | S | S | - |
| 27 | 9582 | R | S | R | R | R | R | R | S | R | R | S | S | - |
| 28 | 9535 | R | R | R | R | R | S | R | S | R | R | R | S | - |
| 29 | 9651 | R | R | R | R | R | S | R | S | R | R | S | S | + |
| 30 | 10024 | R | R | S | R | R | R | R | S | S | R | R | S | - |
| 31 | 10058 | R | R | R | S | S | S | R | R | R | R | R | S | - |
| 32 | 10376 | R | S | S | R | R | S | R | S | R | R | S | S | - |
| 33 | 10389 | R | R | R | R | R | S | R | S | R | R | R | S | + |
| 34 | 10562 | S | R | S | R | R | S | R | R | R | R | R | S | - |
| 35 | 10604 | R | R | R | R | R | R | R | S | R | R | R | S | - |
| 36 | 10623 | R | R | S | R | R | S | R | R | R | R | R | S | + |
| 37 | 10761 | R | R | R | R | R | S | R | R | S | S | S | S | - |
| 38 | 10770 | R | R | R | R | S | S | R | R | R | R | R | S | + |
| 39 | 14075 | R | R | S | R | R | S | R | R | R | R | R | R | - |
| 40 | 40405 | R | S | S | S | R | R | R | S | R | R | S | S | - |
| 41 | 10874 | S | R | R | R | R | S | R | R | S | R | R | S | - |
| 42 | 12732 | R | R | R | S | R | S | R | S | S | R | R | S | + |
| 43 | 11021 | R | R | R | R | R | R | R | R | R | R | R | S | - |
| 44 | 1125 | R | R | S | S | R | S | R | S | R | R | R | S | - |
| 45 | 11360 | R | R | R | S | R | S | R | S | I | R | R | S | + |

+ =lytic activity, - = no activity, R=resistant, I =intermediate=sensitive
